# Impact of updating the non-radiation parameters in the ICRP 103 detriment model

**DOI:** 10.1007/s00411-018-0731-z

**Published:** 2018-01-23

**Authors:** Joachim Breckow, Samaneh Emami, Sara Amalhaf, Arwin Beshgard, Jonas Buermeyer, Kaija Spruck

**Affiliations:** Institute of Medical Physics and Radiation Protection (IMPS), THM, University of Applied Sciences, Giessen, Germany

**Keywords:** ICRP 103, Detriment, Nominal risk coefficient, Cancer lethality, Quality of life, Loss of life expectancy, Severity of damage

## Abstract

The radiation detriment in ICRP 103 is defined as the product of the organ-specific risk coefficient and the damage that may be associated with a cancer type or hereditary effect. This is used to indicate a weighted risk according to the radiation sensitivity of different organs and the severity of damage that may possibly arise. While the risk refers to radiation exposure parameters, the extent of damage is independent of radiation. The parameters that are not affected by radiation are lethality, impairment of quality of life, and reduced life expectancy, which are considered as quantities associated with the severity of disease or damage. The damage and thus the detriment appear to be mostly affected by lethality, which is the quotient of the age-standardized mortality rate to the incidence rate. The analysis of the detriment presented in this paper focuses on the influence of the lethality on the detriment from 1980 to 2012 in the USA and Germany. While the lethality in this period covering more than three decades has decreased approximately linearly by 30% (both USA and Germany), within the same period the detriment declined only by 13% in the USA and by 15% in Germany. If only based on these two countries, an update on the detriment parameters with reference to 2007, when ICRP 103 was released, would result in a reduced weighted risk, i.e. the radiation detriment would be reduced by 10 to 15% from originally 5.7% per Sv for the whole population to roughly 5% per Sv.

## Introduction

To compare the impact of different radiation-induced cancer sites, the detriment model of ICRP 103 (2007) considers not only the organ-specific nominal risk coefficients, i.e. the cancer incidence probability per dose, but also the “severity” of a cancer disease. The ICRP detriment is calculated separately for each specified organ or tissue, which is then combined to render a total detriment for the whole body. The damage or severity is defined as a function of several parameters, including impairment of quality of life, reduced life expectancy, and lethality, which may be associated with a specific cancer disease or hereditary effect. While these parameters are independent of any radiation exposure or radiation effect, they may, however, depend on improvements in cancer diagnoses and treatments, and may thus change with time. It is therefore of some relevance to consider the temporal course of the detriment since the detriment, and in turn the radiation damage, may change with time, even if the radiation risk in terms of the radiation-induced cancer incidence rate remains unaffected.

Furthermore, these damage parameters may possibly change either because of cancer becoming a more common disease within the population, or as a result of the progress in medical diagnostics and treatments that allow patients to survive or at least maintain a higher life quality standard.

The radiation detriment in ICRP 103 (2007) is roughly defined as the product of the (organ specific) nominal risk coefficient and the damage that may be associated with a (organ specific) cancer or hereditary effect. This is used to indicate a weighted risk according to the radiation sensitivity of any different organ and the severity of damage that may possibly arise.

While the risk refers to radiation exposure parameters, the degree of damage is independent of these. The total detriment can be considered as the additional weighted absolute risk per effective dose as a mean value, for the whole population or for the working age population, respectively.

Keeping the general structure of the ICRP detriment model unchanged, in this paper, the temporal development of the model parameters as well as the detriment itself are investigated exemplarily for the USA and for Germany and the consequences are discussed. Some general aspects of a temporal dependence of the radiation detriment may be exemplified through these both countries without considering them as representative for a worldwide cancer development.

## The ICRP 103 detriment model

Even in the case that two different radiation-induced cancer types have identical incidence probabilities, the relevance and severity of the impact arising therefrom may be different. Such differences may be due to different lethality factors, loss of quality of life, or reduced life expectancy, which are considered as quantities associated with the severity of a disease itself or damage caused by the disease. The damage is represented by the confinements that come with cancer and cancer treatment, as well as the possible fatal outcome of the disease.

The definition of the detriment *D*_*T*_ in a tissue *T* along with ICRP 103 (2007) is:

1 $${D_T}={R_T}\, \cdot \,({k_T}+(1 - {k_T})\, \cdot \,{q_T})\, \cdot \,{L_{T,{\text{rel}}}}={R_T}\, \cdot \,{d_T},$$

where *R*_*T*_ represents the (unweighted) incidence risk for cancer in tissue *T* per equivalent dose. These organ-specific nominal risk coefficients *R*_*T*_ are depending on radiation exposure parameters and are not under consideration in the present paper. In the following, these are assumed to be constant and identical with the values given in ICRP 103 (2007) (Table 1).

**Table 1:** Organ-specific input parameters for calculating the total detriment according to the ICRP 103-model (Eq. (1)) exemplified by the values for USA in 2012

Organ/cancer type [ICD-10]	Nominal risk coefficient *R*_*I,T*_ (per 10,000 per Sv)	Lethality factor *k*_*T*_	Quality of life *q*_*T*_(*k*)	Rel. loss of life expect *L*_rel,*T*_	Detriment *D*_*T*_ (per 10,000 per Sv)
Esophagus [C15]	*15*	0.94	0.95	0.95	14.2
Stomach [C16]	*79*	0.46	0.51	0.89	51.7
Colon [C18]	*65*	0.38	0.44	0.84	35.6
Liver [C22]	*30*	0.75	0.78	0.96	27.2
Lung [C34]	*114*	0.83	0.85	0.8	88.9
Bone [C40–C41]	*7*	0.44	0.5	2.1	10.6
Skin [C44]	*1000*	0.0023	0.0023	0.73	3.3
Breast [C50]	*112*	0.16	0.24	1.03	41.7
Ovary [C56]	*11*	0.62	0.66	0.93	8.9
Bladder [C67]	*43*	0.21	0.29	0.64	12.1
Thyroid [C73]	*33*	0.03	0.22	0.85	6.8
Bone marrow [C91–C95]	*42*	0.49	0.54	0.95	30.5
Gonads^a^	*20*	*0.8*	*0.82*	*1.32*	*25.4*
Rest^a^	*144*	*0.49*	*0.54*	*1.03*	*113.5*
			Total detriment		470.4

The values in italic are taken unchanged from ICRP 103 (2007), while the others have been derived with the methods described in the text

^a^Values from ICRP 103 (2007)

The radiation-independent term *d*_*T*_ in Eq. (1) representing the weighting factor for the nominal risk coefficient *R*_*T*_ and in the following termed “severity” of the damage, is determined by three different cancer-related factors (all quantities according to ICRP 103 (2007)):

- The factor *L*_*T*,rel_ represents the relative life lost for a specific cancer type in tissue *T*. It is derived from the quotient of the cancer-type-specific absolute loss of life expectancy to the mean loss of life expectancy averaged over all types of cancer (see Sect. “Material and methods”).
- The lethality factor *k*_*T*_ relates to the probability of a fatal course of a type of cancer and is expressed through the quotient of the age-standardized mortality rate and the age-standardized incidence rate derived from cancer survival data for a specific cancer type *T*.
- The loss of quality of life *q*_*T*_ is defined as a function of *k*_*T*_ :

2 $${q_T}={q_{T,\hbox{min} }}+{k_T}\, \cdot \,(1 - {q_{T,\hbox{min} }}).$$

- This value allows weighting the cancer types that tend to be non-fatal with a minimal quality factor *q*_min_. ICRP 103 gives three different organ-specific values for *q*_min_:
- *q*_min_ = 0 for skin cancer, *q*_min_ = 0.2 for cancer of the thyroid gland, and *q*_min_ = 0.1 for all other cancer types (ICRP 2007).

Some general aspects of the detriment term *d*_*T*_ as a function of lethality are given in the “Appendix”.

## Materials and methods

The time courses of the detriment and its radiation-independent parameters are discussed for the USA and Germany. The calculations are performed on the basis of the detriment model of ICRP 103 (2007) described above. The parameters for the severity *d*_*T*_ are the lethality factor *k*_*T*_ and the relative life lost *L*_*T*,rel_. The mortality and incidence data, that are required for determining these parameters, can be obtained from population-based cancer registries and official population statistics (see below).

For comparison and for exemplarity, calculations in the present paper have been conducted for USA and Germany separately. As an example, the data used for calculating the detriment in USA for the year 2012 are listed in Table 1. The values are given for each type of cancer separately. As in ICRP 103, a minimal loss of live quality *q*_min _= *0.1* has been chosen for all types of cancer, except for skin (*q*_min_ = 0) and thyroid (*q*_min _= 0.2) (2007). In combination with the derived lethality factor, the severity *d*_*T*_ was calculated using Eq. (1).

No updated values for gonads (heritable) and “other solid cancers” (remainder) have been extracted. Instead, the values for these two types of cancer were adopted from ICRP 103 (2007) unchanged and assumed to remain constant over the period from 1980 to 2012.

### Calculation of the severity factor *d*_*T*_

In the following, a stepwise description of the essential values and functions for the calculation of the severity *d*_*T*_ (cf. Eq. (1)) is presented. The calculations are performed separately for each organ or tissue *T*, respectively, and for each year within the time period from 1980 to 2012.

#### Lethality factor *k*_*T*_

The lethality factor *k*_*T*_ is defined as the quotient of the age-standardized mortality rate for cancer site *T* and the corresponding age-standardized incidence rate.

The age-specific mortality or incidence rate *r*_*i*_(*t*) is estimated by the relation:

3 $${r_i}(t)=\frac{{{n_i}(t)}}{{\Delta A\, \cdot \,\Delta t}}\, \cdot \,\frac{{\Delta A}}{{{N_i}(t)}}=\frac{{{n_i}(t)}}{{{N_i}(t)\, \cdot \,\Delta t}},$$

where *n*_*i*_(*t*) is the number of cases or deaths, respectively, at the time of observation *t* within age group *i* of width ∆*A* and within the observation duration of width ∆*t. N*_*i*_(*t*) is the mean number of individuals within age group *i* of the resident population that are alive at time of observation *t* (i.e. that are “at risk”). Calculations in this paper are performed on age groups of the width ∆*A* = *5a* and for every single year, i.e. ∆*t* = *1a*, in the time span from 1980 to 2012.

The cumulative mortality or incidence rate *P*(*t*) results from the summation over all age groups *i* up to an age group *m* that corresponds to a specified upper age *A*_*m*_ (normally the oldest age group):

4 $$P(t)=\sum\limits_{{i=1}}^{m} {{r_i}(t)\, \cdot \,\Delta A} =\sum\limits_{{i=1}}^{m} {\frac{{{n_i}(t)}}{{{N_i}(t)}}\, \cdot \,\frac{{\Delta A}}{{\Delta t}}} .$$

The cumulative rate *P* is a convenient form of direct age standardization, i.e. all considered age groups up to *m* are equally weighted and no assumption for an age distribution is required. In general, however, an age standardization is a weighted sum of the age-specific rate according to a specified population-related age distribution. For the present paper, the age-specific survival functions *S*_*i*_(*t*) for each country and each year under investigation are chosen as appropriate weighting functions for the weighted cumulative mortality rate ^*M*^*P*_*S*_ and the weighted cumulative incidence rates ^*I*^*P*_*S*_ :

5 $${}^{M}{P_{\text{S}}}(t)=\frac{{\Delta A}}{{\Delta t}}\, \cdot \,\sum\limits_{{i=1}}^{m} {\frac{{{}^{M}{n_i}(t)}}{{{N_i}(t)}}\, \cdot \,{S_i}(t)} \quad {\text{and}}\quad {}^{I}{P_{\text{S}}}(t)=\frac{{\Delta A}}{{\Delta t}}\, \cdot \,\sum\limits_{{i=1}}^{m} {\frac{{{}^{I}{n_i}(t)}}{{{N_i}(t)}}\, \cdot \,{S_i}(t)} ,$$

where ^*M*^*n*_*i*_ and ^*I*^*n*_*i*_ are the number of deaths and incidence cases, respectively, in age group *i*. ^*M*^*P*_S_ and ^*I*^*P*_S_ are the corresponding age-standardized cumulative mortality rates and incidence rates, respectively.

The survival function *S*_*i*_(*t*) describes the probability for surviving from the time of birth up to the age of interest *A* according to age group *i* and at the time of observation *t*. It can be calculated with the cumulative mortality rate ^*M*^*P*_all_ from the entirety of all causes of death for the year *t* of interest:

6 $${S_i}(t)=\exp \;\left( { - {}^{M}{P_{{\text{all}}}}(t)} \right)=\prod\limits_{{k=1}}^{i} {\exp \left( { - \frac{{\Delta A}}{{\Delta t}}\, \cdot \,\frac{{{}^{M}{n_{k,{\text{all}}}}(t)}}{{{N_k}(t)}}} \right)} ,$$

where ^*M*^*n*_*k*,all_ is the number of deaths in age group *k* from all causes of death.

For a rigorous derivation see for example Lawless (2003) or Jensen et al. (1991).

For the calculation of the lethality factor *k*_*T*_, the age-standardized cumulative mortality rates and incidence rates must be calculated according to Eq. (5) for each tissue *T*. The lethality factor for tissue *T* at observation time *t* is

7 $${k_T}(t)=\frac{{{}^{M}{P_{S,T}}(t)}}{{{}^{I}{P_{S,T}}(t)}}.$$

If the lethality factor *k*(*t*) for the entirety of all cancers is required, Eq. (7) yields

8 $$k(t)=\frac{{{}^{M}{P_S}(t)}}{{{}^{I}{P_S}(t)}}.$$

#### Relative life lost *L*_*T*,rel_

The relative life lost *L*_*T*,rel_ is the life lost *L*_*T*_ due to the cancer in tissue *T* relative to normal life expectancy, expressed relative to the average ${\overline {L} _T}$ over all cancers. At any age *A* of life, there is an expectation value for how many time is expected still being alive. This is the mean probability of survival *a*_*i*_(*t*) at attained age *A* corresponding to age group *i* at time of observation *t*. The probability of survival is the sum of the age-specific survival function *S*_*k*_(*t*) (cf. Eq. (6)) over all age groups *k*, starting at the attained age *A* corresponding to age group *i*, and normalized to the probability *S*_*i*_ for surviving up to age group *i*, e.g. Lawless (2003):

9 $${a_i}(t)=\frac{{\sum\nolimits_{{k=i}}^{m} {{S_k}(t)\, \cdot \,\Delta A} }}{{{S_i}(t)}}.$$

Any cause of death (e.g. cancer in tissue *T*) in a population give rise to a mean age at death ^*M*^*A*_*T*_(*t*). For the age group *i* that corresponds to the mean age at death ^*M*^*A*_*T*_(*t*) the probability of survival *a*_,*i*_(*t*) according to Eq. (9) is calculated. If dying at age ^*M*^*A*_*T*_ due to a specified cause of death the statistically remaining span of life cannot be experienced anymore. This life expectancy (probability of survival) *a*_*i,T*_(*t*) at age ^*M*^*A*_*T*_, approximately corresponds to the absolute life lost *L*_*T*_(*t*) due to a specified cause of death (e.g. due to cancer type *T*):

10 $${a_{i,T}}(t)={L_T}(t).$$

For each type of cancer *T* and for each year *t* considered the mean age at death ^*M*^*A*_*T*_(*t*) is calculated by means of the age-specific mortality rate ^*M*^*r*_*i,T*_(*t*) weighted by the age-specific survival function *S*_*i*_(*t*) (cf. Eqs. (4) and (5)) and is approximated by

11 $${}^{M}{A_T}(t)=\frac{{\sum\nolimits_{{i=1}}^{m} {{}^{M}{r_{i,T}}(t)\, \cdot \,{S_i}(t)\, \cdot \,{{\bar {A}}_i}} }}{{\sum\nolimits_{{i=1}}^{m} {{}^{M}{r_{i,T}}(t)\, \cdot \,{S_i}(t)} }}\quad \to \quad {\text{age}}\,{\text{group}}\,i,$$

where ${\bar {A}_i}$ is the mean age of age group *i*.

The relative life lost *L*_*T*,rel_(*t*) is then calculated according to

12 $${L_{T,{\text{rel}}}}(t)=\frac{{{L_T}(t)}}{{{{\bar {L}}_T}(t)}}=\frac{{n\, \cdot \,{L_T}(t)}}{{\sum\nolimits_{{T=1}}^{n} {{L_T}(t)} }},$$

with the sum representing the overall life lost due to all *n* cancer types *T*. The relative life lost *L*_*T*,rel_ allows to compare the impact of each different cancer type or tissue, respectively, with respect to the life time that is lost due to the disease.

Note, that all quantities in Sect. 3.1 are independent of any radiation exposure.

### Data

The mortality and incidence data required for the calculations have to comply with special demands. To calculate age-specific rates, the data on incidence and mortality have to be provided for each age, or at least within sufficiently narrow sized age groups. In this paper, the age groups have a width of 5 years. Furthermore, the data need to provide the information for each type of cancer listed in ICRP 103 (2007). To satisfy the formal demands, the necessary data had to be obtained from different sources or, occasionally, had to be estimated.

The cancer-specific mortality and incidence data for the USA were provided by the SEER-tables of the US National Cancer Institute (NCI 2016) and by the Centres for Disease Control and Prevention (CDC) of the US Dept. Health and Human Services (CDC 2016). An exception had to be made for the incidence data for skin cancer (see below). The skin mortality data were included in the data provided by the SEER-tables, in the category of the non-melanoma skin cancer.

The cancer-specific incidence data for Germany had to be obtained from different sources to cover the whole time span considered. From 1980 to 1998, the incidence data were obtained from the Cancer Registry of the federal state (Bundesland) Saarland (2016). Since no source provided this information on a federal level, the Saarland data were assumed representative for the entire country. The cancer-specific incidence data from 1999 to 2012 were provided by the German Centre for Cancer Registry Data of the Robert-Koch-Institute (RKI 2016) for all types of cancer except the skin cancer. Skin cancer data from 1980 to 1998 were taken from the Cancer Registry of Saarland (2016), which registers the basal cell carcinoma (BCC) and the squamous cell carcinoma (CSCC) of the skin. For the years from 1999 to 2012, the data were provided by the Association of Populations-based Cancer Registries in Germany (GEKID 2016). The cancer-specific mortality information was obtained from the German Information System of Federal Health Monitoring (GBE 2016) for the whole time span from 1980 to 2012. Populations-based data came from the GENESIS-database of the German Federal Statistical Office (GENESIS 2016).

Different to the data used in ICRP 103 (2007) which reach up to age group 90 + (90 years and above), the data in this paper were up to age group 85 + (85 years and above). The goal of the present calculations predominately is to be consistent with each other in each dataset, rather than to produce values directly comparable to the ICRP data.

#### Skin cancer, other solid cancers (remainder), gonads (heritable)

For mortality data, many international cancer registries report a skin cancer group “other skin cancer” with ICD10 C44 that includes all non-melanoma skin cancers, particularly basal cell carcinoma of the skin (BCC) and squamous cell carcinoma of the skin (SCC). However, BCC usually is not identified separately. Due to the very low mortality of BCC (as well as SCC) only a very small detriment arises, leading finally—despite the extraordinary high risk coefficient for skin in ICRP 103—to a very small contribution of skin cancer to the total detriment. Thus, for the purpose of the present paper the skin cancer subgroup “other skin cancer” (ICD10 C44) is considered as a sufficient proxy for BCC, or respectively, for the tissue “skin” according to ICRP 103 (2007).

Due to potential multiple diagnoses and other uncertainties in the registration procedures, most international cancer registries provide no detailed incidence data for non-melanoma skin cancers (including BCC and SCC). In the same way, the SEER-tables of the US Dept. Health and Human Services as well as the German Robert-Koch-Institute report no incidence rates for BCC or SCC. In contrast, mortality data for non-melanoma skin cancer (ICD10 C44) are available (CDC 2016; RKI 2016).

Nevertheless, to obtain incidence data for the non-melanoma skin cancer in the USA, a rough approximation based on an interpolation procedure performed by Karia et al. (2013) is performed. Their calculations were based on cohort surveys of age-adjusted incidence rates for SCC. The surveys were sorted into two different “sun zones” of the USA covering the time range from 1971 to 1999. By applying a linear regression model, a linear temporal increase of the incidence rate up to 2012 was obtained for each zone. To gain a rough representative for the USA, the rates calculated for each zone were combined to an overall average incidence rate for SCC. Thus, due to the assumed linearly increasing temporal course of the incidence rate an approximation for each year in the time range 1980–2012 regarded in the present paper could be achieved.

For estimating the incidence rates for BCC it is assumed that the temporal course follows the incidence rates of SCC with a constant proportional factor of approximately equal to 1, i.e. the incidence rates for both skin cancer types are nearly the same. With these assumptions, the combined incidence rate for BCC and SCC represents a sufficient proxy for the skin cancer subgroup “other skin cancer” (ICD10 C44) to calculate the mortality factor *k* for the USA with the quotient of the skin mortality rate and the skin incidence rate (cf. Eq. (7)).

The cancer registry of the German Federal state (Bundesland) of Saarland registers age-specific incidence rates of the skin subgroup “other skin cancers” (including BCC and SCC). These are considered representative for the whole country and are used for the calculations of the skin cancer detriment for Germany in the time period from 1980 to 1998 without further adjustments. For the period from 1999 to 2012 estimated incidence data for “other skin cancers” (including BCC and SCC) in Germany are available by the Association of Populations-based Cancer Registries in Germany (GEKID 2016).

For each of the 12 single cancer sites with a detriment calculation in ICRP 103 (2007) the incidence and mortality data necessary for the calculations in the present paper could be obtained from population and cancer registries mentioned above. For the two remaining tissues groups “Other solid cancers (remainder)” and “Gonads (heritable)” no calculations are performed. For these groups all parameters are taken unchanged from ICRP 103 (2007) and are considered to remain constant over the entire time period from 1980 to 2012. In a last step, the total detriment is derived as the sum over all *k* = *14* organs *T* considered by ICRP 103. As an example, in Table 1 this procedure is shown for the USA in 2012.

## Results

### Temporal development of cancer lethality

**Fig. 1:**
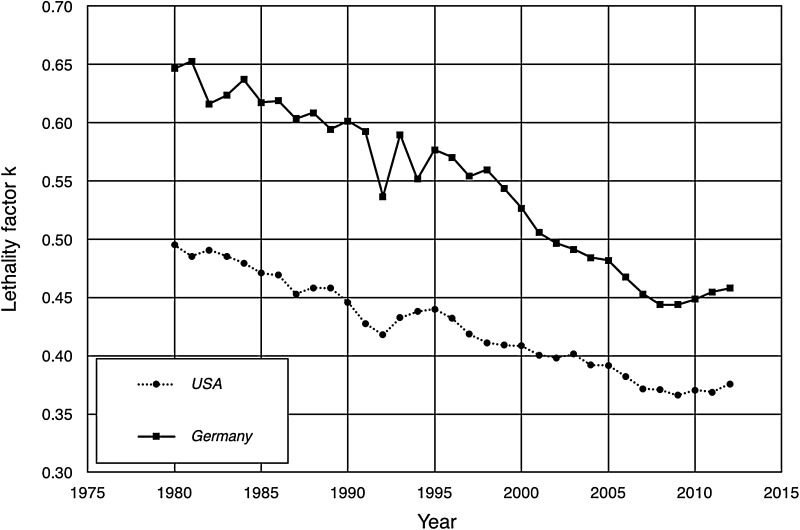
Temporal course of the cancer lethality factor *k* due to all cancer types in USA (dotted line) and Germany (solid line) between 1980 and 2012

The combined lethality factor *k*_*T*_(*t*), calculated separately for each of the 12 cancer sites considered (cf. Table 1), derived from the age-standardized incidence rates ^*I*^*P*_*S*_(*t*) and mortality rates ^*M*^*P*_*S*_(*t*) according to Eq. (8), is shown in Fig. 1 as functions of time from 1980 to 2012, separately for USA and Germany. Starting at a value of about 0.5 for USA and about 0.65 for Germany, both datasets show a strong decrease during the time period considered. The decrease is about 30% both in Germany and USA, while for 2012 the overall lethality for all types of cancer is 0.38 for USA and 0.46 for Germany, respectively.

### Temporal development of the detriment

Parallel to the temporal course of the lethality, using the methods described above, the total detriment, calculated with Eq. (1) separately for each organ *T* from 1980 to 2012, is analysed. The organ-specific unweighted nominal risk coefficients *R*_*T*_ are assumed to be constant in time and identical with the values given in ICRP 103 (2007) (Table 1).

**Fig. 2:**
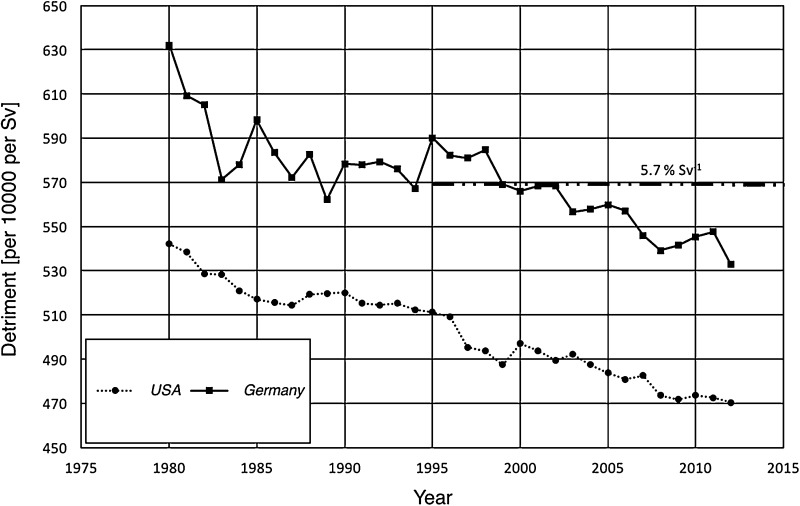
Temporal course of the total detriment as the sum of all organ-specific detriments according to Eq. (1) in USA (dotted line) and Germany (solid line) between 1980 and 2012. For comparison, the ICRP-103 total detriment for the whole population of 5.7%·Sv^− 1^ (ICRP 2007) is given as the horizontal dash-dotted line

The detriment is calculated for each type of cancer separately and then summed up to a total detriment of the body. This procedure was repeated for each considered year between 1980 and 2012. The results are shown in Fig. 2 where the derived detriment for USA and Germany is plotted as functions of time. Both datasets show a decrease with time, while the detriment in Germany is about 10% higher than the detriment in the USA at all times. However, the slope of the decline is similar with 15% in Germany and 13% in the USA over the considered 32 years. For both, USA and Germany, the decrease in lethality over the considered time span is more than two times the decrease in detriment.

When comparing the values of successive years, the data for Germany have with about 5% a slightly larger scatter than the values from the USA. The detriment in Germany starts with a value of about 630 × 10^− 4^ Sv^− 1^ in 1980 and decreases down to about 530 × 110^− 4^ Sv^− 1^ in 2012, while in the USA it starts at about of 540 × 110^− 4^ Sv^− 1^ in 1980 and decreases to below of 470 × 110^− 4^ Sv^− 1^ in 2012. With some fluctuations, the detriment in Germany matches the ICRP 103 (2007) value of 570 × 110^− 4^ Sv^− 1^ (5.7% per Sv) fairly well between 1985 and 2005.

### Examples for temporal development in organ-specific detriments

**Fig. 3:**
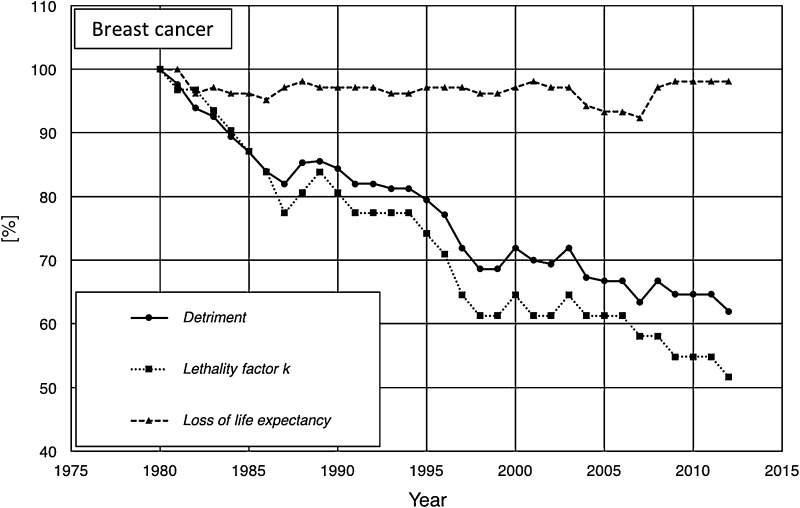
Temporal course of lethality (dotted line), loss of life expectancy (dashed line), and detriment (solid line) for breast cancer in the USA between 1980 and 2012. All data are given as relative values with reference to 1980 (100%)

**Fig. 4:**
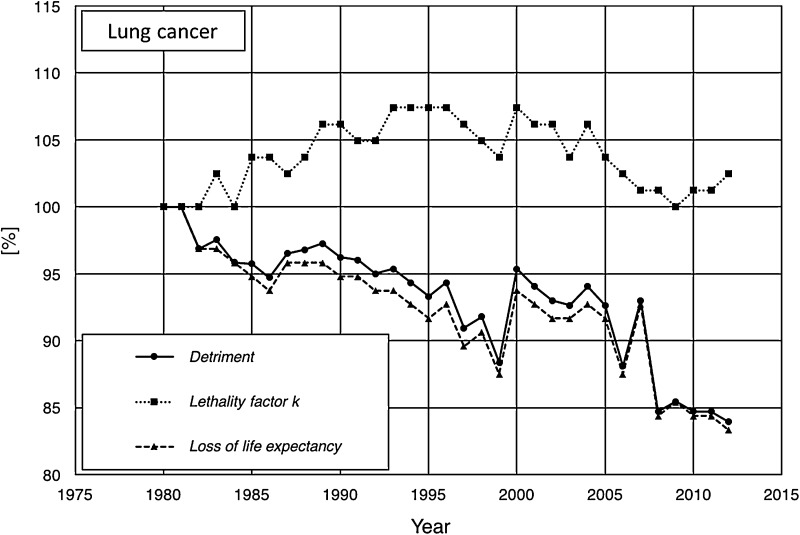
Temporal course of lethality (dotted line), loss of life expectancy (dashed line), and detriment (solid line) for lung cancer in the USA between 1980 and 2012. All data are given as relative values with reference to 1980 (100%)

Two opposing examples for the relationship between lethality, loss of life expectancy, and detriment for a specific type of cancer are given in Fig. 3 (breast cancer, USA) and Fig. 4 (lung cancer, USA). For both types of cancer, the detriment is decreasing over the considered period of time. To reveal the different influences to the course of the detriment, the relative temporal development is depicted (with 1980 as reference). The relative lethality of breast cancer (Fig. 3) shows a similar trend of decreasing slope as the corresponding detriment, while the relative loss of life expectancy (relative to 1980) differs significantly from the time course of the corresponding detriment. In contrast, comparing the time courses of the detriment, lethality and loss of life expectancy for lung cancer (Fig. 4) here apparently the decreasing loss of life expectancy is the dominating cause for the temporal development of the detriment, while the lethality shows a different temporal course. Thus, both, the loss of life expectancy and the lethality may have—separately or combined—an impact on the damage *d*_*T*_ (cf. Eqs. (1) and (13)), and can hence lead to a variation of the detriment.

**Fig. 5:**
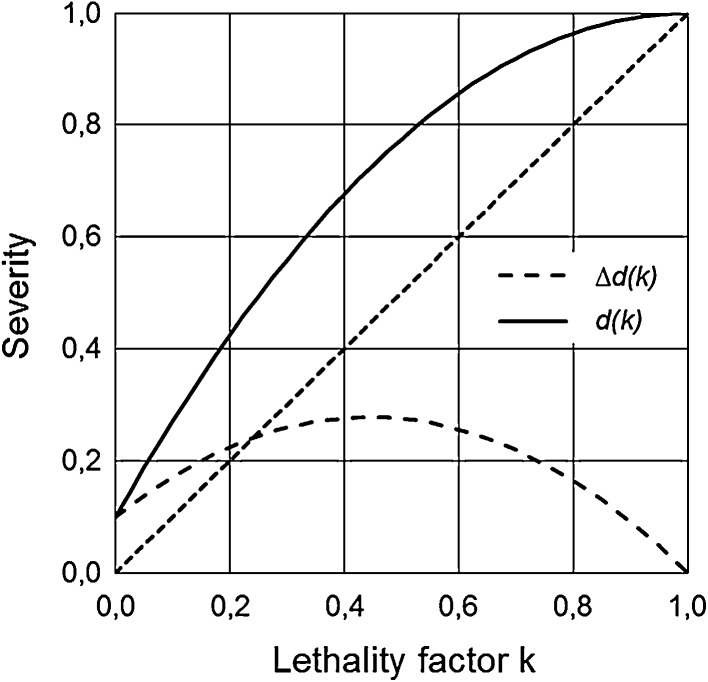
Severity term *d*(*k*) (solid line) and the non-lethal part of life quality loss Δ*d*(*k*) (dashed line) as functions of the lethality factor *k* according to Eqs. (13) and (14). The relative loss of life expectancy in Eq. (13) is set to *L*_rel_=*1*. The minimum loss of life quality is *q*_min_ = *0.1*. For comparison, the angle bisecting line, i.e. the lethality factor *k* itself, is shown as fine-dashed line

**Fig. 6:**
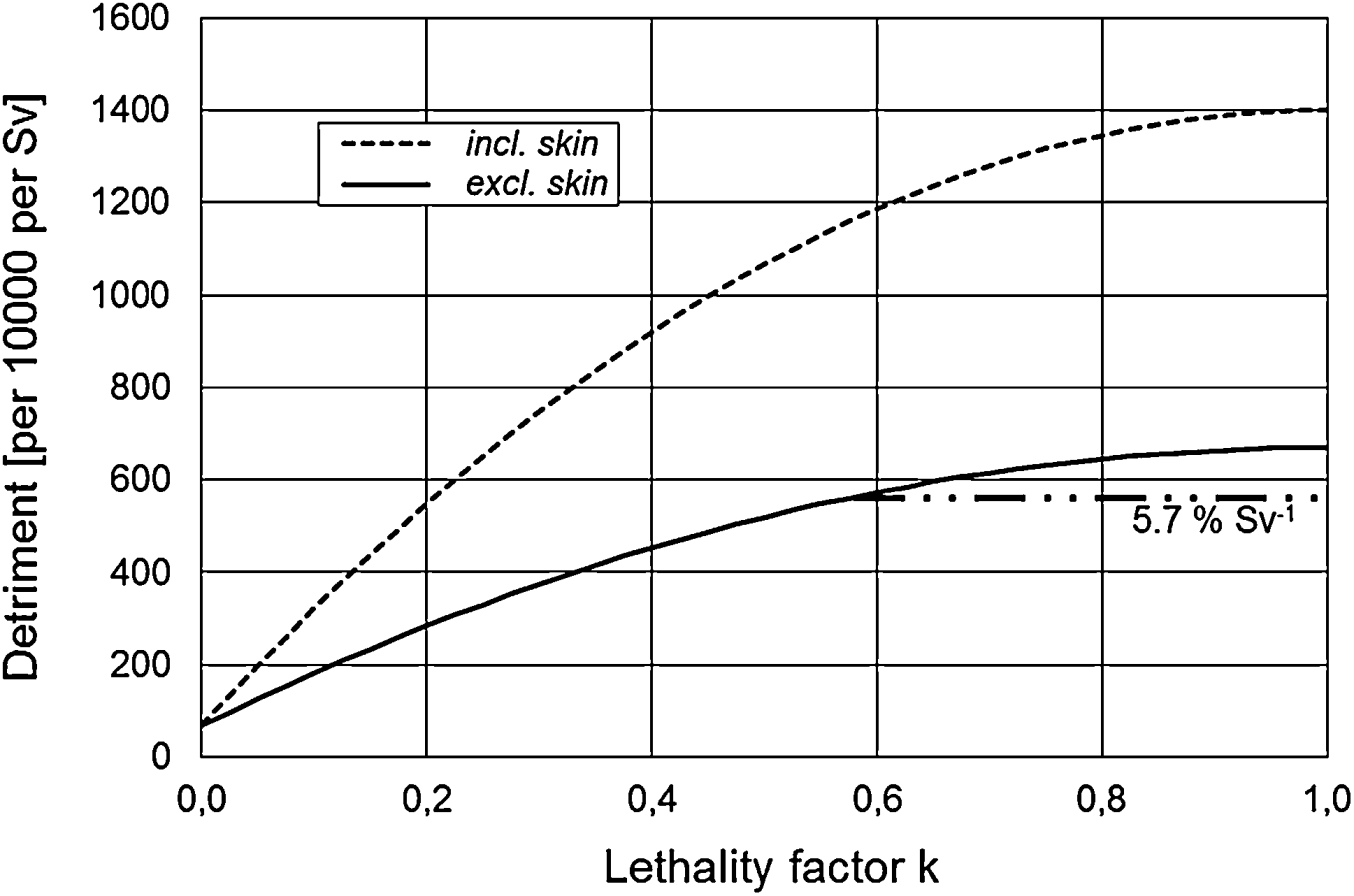
The total detriment *D*(*k*) as a function of the lethality factor *k* according to Eq. (16). The organ-specific nominal risk coefficients *R*_*T*_, the lethality factors *k*_*T*_, and the relative losses of life expectancy *L*_rel,*T*_ are taken from Table 1 (USA, 2012). The total detriment is shown for two cancer entities, the sum of all cancer types (all cancers incl. skin, dashed line) and the sum of all cancer types excluding skin (solid line). For comparison, the ICRP total detriment for the whole population of 5.7%·Sv^− 1^ (2007) is given as the horizontal dash-dotted line

## Discussion

It is of remarkable meaning that not only radiation parameters, such as the organ-specific risk coefficients enter into the definition of the detriment of ICRP, but also some general health parameters, such as lethality or live loss. These parameters characterize the “amount” or the “severity” of a radiation-induced effect, while they are itself independent of any radiation exposure. In fact, these parameters are subject to a development over time, reflecting both the general development of cancer in the population and the advances in medical diagnostics and therapy. Due to these developments, if the amount of damage, and thus, the detriment decreases in a temporal development, the radiation risk is reduced as well, and that without the mechanisms of the underlying radiation effects themselves are being changed or affected. The “radiation effect” defined on the basis of the ICRP detriment model is thus dependent on the development of cancer in the population and of the medical progress. Looked at it superficially, this fact might seem somewhat unreasonable. It is questionable as to why it should be allowed to define a radiation risk that is influenced or weighted by non-radiation health parameters. Furthermore, it is unclear whether the relevance and impact of radiation effects, in addition to the perception of the public, are indeed dependent on medical progress.

The procedure of setting dose limits in radiation protection usually requires a linking of “dose” (e.g. effective dose) and “radiation effect” (e.g. detriment). Thus, a dose limit would indicate a maximum tolerable risk, i.e. a tolerable detriment. If in the course of a reduction in the cancer mortality rate, the detriment would decrease as well (with unchanged organ-specific risk coefficients), a higher dose limit would correspond to the same given (and unchanged) maximum tolerable risk. Such a consideration would have significant implications for the concepts of radiation protection, and in particular, for the communication and perception of radiation risks in the public debate.

Among the parameters included in the ICRP detriment system, the dose and dose-rate effectiveness factor (DDREF) plays an important role (Rühm et al. 2016). In a recent recommendation on the DDREF, the German Commission on radiological protection (SSK) recommends to adjust the DDREF according to the newest scientific findings, and if necessary, to abolish the DDREF (SSK 2014). Furthermore, due to its relevance for the risk assessment and the implications for radiation protection, the SSK recommends adapting all other parameters that are components of the detriment during this adjustment, too. Although the SSK was aware that these effects are totally independent, the consideration was that the abolition of the DDREF would result in an increase of the assessment of radiation effects, on the one hand, adjusting the other detriment parameters, on the other hand, would have some compensatory effect. However, the findings presented here indicate that even with a sharp decline in the mortality rate from cancer (about 30% in the period of over 30 years) the detriment decreases only by 10–15%.

In this paper, only two countries are analysed, both featuring a good healthcare standard in 1980 and improving ever since. The temporal development of the analysed non-radiation parameters can be quite different and possible also contrary in other countries. Since the ICRP defines a detriment on the basis of a worldwide dataset, the resulting effect of an update of these parameters on the detriment can be different from these findings. However, the presented results could provide a sound basis to understand origin and effect of possibly observed temporal developments even on a worldwide basis.

## Appendix

An analysis of the behaviour of the detriment calculation formulas according to values of non-radiation-related parameters might be of some interest to judge the impact that variations of these parameters might have. These results do not depend on data used in Sect. “Results”.

### Variations in lethal and non-lethal damages

At first, the dose-independent severity *d*(*k*) in Eq. (1) is examined on a theoretical basis,

13 $$d(k)=(k+(1 - k)\, \cdot \,q)\, \cdot \,{L_{{\text{rel}}}}.$$

For these calculations, the relative loss in life expectancy *L*_rel_ is set to *1*. The remaining function of *d*(*k*) is a composition of the lethality factor *k* itself and a non-lethal part of life-quality loss, denoted as Δ*d*(*k*):

14 $$d(k)=k+\Delta d(k)\quad {\text{with}}\;\Delta d(k)=(1 - k)\, \cdot \,q.$$

In Fig. 5, both functions are plotted against *k*. In this case, a value for minimum loss of life quality *q*_min_ = *0.1* was chosen which corresponds to the value used by ICRP 103 (2007) for all types of cancer except for skin and thyroid.

The function *d*(*k*) and its non-lethal portion Δ*d*(*k*) are calculated for values of *k* starting from 0 for (hypothetical) non-lethal types of cancer, up to a value of 1 for types of cancer which always have a lethal course. The lethal part of *d*(*k*), i.e. the lethality factor *k* itself, produces an angle bisecting line. For *k* = *0* and *k* = *1*, the non-lethal function Δ*d*(*k*) arrives at its extreme values of:

15 $$\Delta d(0)={q_{\hbox{min} }}\quad {\text{and}}\quad \Delta d(1)=0.$$

Starting from the minimum value of *q*_min_ = *0.1*, the non-lethal term Δ*d*(*k*) increases towards higher values of *k* in a soft slope. The maximum of 0.28 is reached at a lethality of *k* = 0.45. Towards higher values of *k*, Δ*d*(*k*) decreases again and arrives at its minimum value of 0 at the maximum lethality of *k* = 1.

The entire severity function *d*(*k*) also features two extreme values of *d*(0) = *q*_min_ and *d*(1) = 1. Hence, *d*(*k*) starts for *k* = 0 at the same minimum value of *q*_min_ = 0.1 and increases in a parabolic curve towards higher values of *k*. For *k* = 1, *d*(*k*) reaches its maximum value of 1, which is, for this given minimum loss of life quality, ten times the value at *k* = 0.

By comparing the angle bisecting line for the lethal term of *d*(*k*) and the non-lethal term Δ*d*(*k*), it is noticeable that, starting from a value for *k* of about 0.25, the function *d*(*k*) is dominated by the lethal part *k*. On the other hand, a variation in *k* has a stronger effect on the severity function *d*(*k*) at smaller values for *k*, than for higher values of *k*.

### Detriment as a function of lethality

The detriment of ICRP 103 (2007) predominately depends on the cancer lethality *k*_*T*_ (Eq. (1)), where, in turn, the reduction of life quality *q*_*T*_ depends on the cancer lethality as well. Thus, in a further step, it is examined in which way the detriment changes if the lethality is varied. The variation of the lethality in this sense is performed to study the entire theoretical lethality range including some hypothetically extreme or typical situations and to get a rough figure of the detriment as a function of lethality. For this purpose, it is assumed that all parameters other than the lethality remain constant, particularly the organ-specific unweighted nominal risk coefficients *R*_*T*_ (taken from ICRP 103 (2007)) as well as the organ-specific relative loss of life expectancy *L*_*T*,rel_ (reference: USA, 2012, cf. Table 1). The lethality factor *k* is the variable of the detriment function and is identical for all tissues. With these assumptions, the total detriment $D(k)=\sum\nolimits_{T} {{D_T}(k)}$ is considered as a function of lethality in the range of *k* = 0 … 1 [cf. Eq. (1)]:

16 $$D(k)=\sum\limits_{T} {{R_T}\, \cdot \,{L_{T,{\text{rel}}}}} \, \cdot \,\left( {{q_{T,\hbox{min} }}+2k\, \cdot \,(1 - {q_{T,\hbox{min} }}) - {k^2}\, \cdot \,(1 - {q_{T,\hbox{min} }})} \right).$$

With Eq. (16) the detriment is performed for two cancer entities, the sum of all cancer sites (all cancers incl. skin) and the sum of all cancer sites excluding skin (Fig. 6). Since cancer of the skin in contrast to all other cancer types has no contribution at all at *k* = *0* (nobody dies for cancer), and it has a large contribution at *k* = *1* (everybody dies if comes down with cancer), a considerable difference occurs between the detriment with and without skin cancer (cf. Fig. 6). The reason is the extraordinary high incidence, and thus, large risk coefficient for skin cancer [${R_{{\text{Skin}}}}=1000 \times {10^{ - 4}}\, \cdot \,{\text{S}}{{\text{v}}^{ - 1}}$, Tab.A.4.1. in ICRP (2007)].

For *k* = *0* and *k* = *1*, respectively, it follows from Eq. (16):

17 $$D(0)=\sum\limits_{T} {{R_T}\, \cdot \,{L_{T,rel}}\, \cdot \,{q_{T,\hbox{min} }}\quad {\text{and}}\quad D(1)=\sum\limits_{T} {{R_T}\, \cdot \,{L_{T,{\text{rel}}}}} } .$$

In the hypothetical case that all cancers are fatal (*k* = *1*), the total detriment predominately is determined by the cancer-incidence probability per dose, i.e. the risk coefficient *R*_*T*_. In the case that no cancer is fatal (*k* = *0*), the detriment is lower by the factor of *q*_min_. Since most cancer sites (except skin and thyroid gland) are associated with *q*_min_ = 0.1, the detriment (excluding skin) for *k* = 1 is approximately ten times higher than for *k* = *0* (cf. Fig. 6; the included cancer of the thyroid gland with *q*_min_ = 0.2 is of minor impact). In contrast, the detriment solely due to skin cancer is (cf. Table 1 exemplified by USA, 2012)

18 $${D_{{\text{Skin}}}}(0)=0\quad {\text{and}}\quad {D_{{\text{Skin}}}}(1)=1000\, \times \,{10^{ - 4}}\, \times \,S{v^{ - 1}}\, \times \,0.73=730\, \times \,{10^{ - 4}}\,{\text{S}}{{\text{v}}^{ - 1}}.$$

As can be seen in Fig. 6 a, hypothetical reduction of lethality, of say 20%, would result in a relatively low reduction of detriment if the lethality is high, whereas it would lead to a higher reduction if the lethality is low. This corresponds to the observed dependencies of the function *d*(*k*) from the lethality factor *k*.

ICRP 103 (2007) gives a detriment-adjusted nominal risk factor for stochastic effects of 5.7% per Sv for the whole population. The theoretical detriment excluding skin cancer matches this value approximately for *k* = *0.6*, corresponding roughly to the fact that about half of patients diagnosed with any type of cancer will die on it.
